# Safety and oncological outcome of early intraoperative intravesicle mitomycin C vs. deferred instillation in patients receiving robot-assisted radical nephroureterectomy

**DOI:** 10.3389/fsurg.2024.1366982

**Published:** 2024-04-25

**Authors:** Sheng-Feng Chou, Wei-Ching Lin, Han Chang, Chi-Ping Huang

**Affiliations:** ^1^ Department of Urology, China Medical University Hospital, Taichung, Taiwan; ^2^ School of Medicine, China Medical University, Taichung, Taiwan; ^3^ Department of Radiology, China Medical University Hospital, Taichung, Taiwan

**Keywords:** radical nephroureterectomy (RNU), mitomycin-C (MMC), recurrence, adverse event (AE), upper tract urothelial carcinoma (UTUC)

## Abstract

**Introduction:**

Radical nephroureterectomy with concurrent bladder cuff excision (RNUBCE) is the gold standard surgical approach for high-risk primary upper tract urothelial carcinoma (UTUC). Given the notably high incidence of bladder tumor recurrence following this procedure, this study aimed to evaluate the effect and safety of intraoperative mitomycin-C (MMC) instillation vs. deferred instillation on overall oncological outcomes following robot-assisted RNUBCE.

**Methods:**

This is a retrospective chart review study. Patients with non-invasive (N0, not T3/T4) UTUC who underwent robotic RNUBCE combined an intraoperative MMC instillation or a deferred MMC instillation after surgery at a medical center in Taiwan between November 2013 and June 2020 were eligible for inclusion. Patients with prior bladder UC, carcinomas of other origins, received neoadjuvant chemotherapy, and had undergone kidney transplantation were excluded. All surgeries were executed by a single surgical team under the guidance of the same surgeon. The primary outcomes was the risk of bladder tumor recurrence between patients received intraoperative (IO) vs. deferred MMC instillation postoperatively (PO) during one-year follow-up. The secondary outcome was postoperative adverse events assessed by the Clavien–Dindo classification. Univariate and multivariable Cox regression analyses were performed to determine the associations between study variables and the outcomes.

**Results:**

A total of 54 patients were included in the analysis. 12 (22.2%) patients experienced a bladder tumor recurrence during follow-up (IO: 7.7%, PO: 35.7%, *p* < 0.021). After adjustment in the multivariable, intraoperative MMC instillation was significantly associated with lower risk of bladder recurrence [adjusted hazard ratio (aHR) = 0.15, 95% CI: 0.03–0.81, *p* = 0.028]. No MMC-related Clavien–Dindo Grade III–IV adverse events were found in either group.

**Conclusion:**

IIntraoperative MMC instillation is safe and associated with a lower bladder tumor recurrence risk in patients undergoing robotic RNUBCE for UTUC than deferred instillation. Future large, prospective studies are still warranted to confirm the findings.

## Introduction

Upper tract urothelial carcinoma (UTUC) is a urothelial malignancy involving the renal pelvis or ureter. It is a relatively rare disease and accounts for approximately 5%–10% of all UCs (1). Radical nephroureterectomy with a bladder-cuff excision (RNUBCE) is the gold standard surgical approach for high-risk UTUC across the majority of patients (2). The risk of tumor recurrence in the bladder after the surgery is considerable, estimated at 22%–47% (3). The initial intravesical relapse typically occurs within the first two years following the treatments, and recurrence is regarded as common with a lifelong risk (3).

In superficial bladder cancers, a prospective trial has revealed that the administration of immediate mitomycin-C (MMC) within 24 h after transurethral resection of bladder tumor (TURBT) outperformed the deferred application of MMC over two weeks (4). Additionally, apart from the commonly used MMC, other intravesical chemotherapeutics like epirubicin and pirarubicin (THP) have exhibited effectiveness in reducing the risk of recurrence (5–7). It has been demonstrated that single-dose intravesical chemotherapy can successfully prevent bladder cancer recurrence following NU by lowering the intravesical recurrence rate (8). However, concerns about the risk of adverse events from extravasation of these intravesical agents still exist (6, 9, 10). There is uncertainty regarding the optimal timing for initiating intravesical chemotherapeutic agents, whether in the early stages of immediate postoperative care or after confirming the cystogram, which indicates the absence of extravasation (6). The absence of a specified timing for the planned instillation has left a gap in understanding, and its potential to adversely impact the efficacy of reducing bladder recurrence remains uncertain (8).

In this study, we conducted a retrospective evaluation to scrutinize the oncological results and safety profiles associated with the prompt administration of a single dose of MMC into the bladder during the execution of a standardized robot-assisted radical nephroureterectomy (RARNU) technique. Our analysis focused on the bladder tumor recurrence (BTR) for one year and short-term adverse events.

## Methods

### Patient selection

Patients' electrical medical records at a tertiary referral center in Taiwan were retrospectively reviewed between November 2013 and June 2020. The inclusion criteria were: patients with non-invasive disease (N0, not T3/T4) UTUC who underwent robotic RNUBCE combined early intraoperative MMC instillation or deferred instillation. The exclusion criteria were patients with prior bladder UC, carcinomas of other origins, who received neoadjuvant chemotherapy, and who had undergone kidney transplantation. Patients were categorized into two groups based on the timing of MMC administration: patients received early MMC instillation intraoperatively (i.e., the IO group) and deferred MMC instillation at least 24 h postoperatively (i.e., the PO group).

The study did not employ random allocation; instead, treatment modalities were determined by temporal factors. Specifically, IO treatments were administered after February 2018, whereas prior to this, PO treatments were used. At that time, the decision to use IO instead of PO was based on emerging research findings in the literature suggesting that early instillation could potentially prevent bladder recurrence following transurethral resection of a bladder tumor.

### Surgical technique

**Figure 1:**
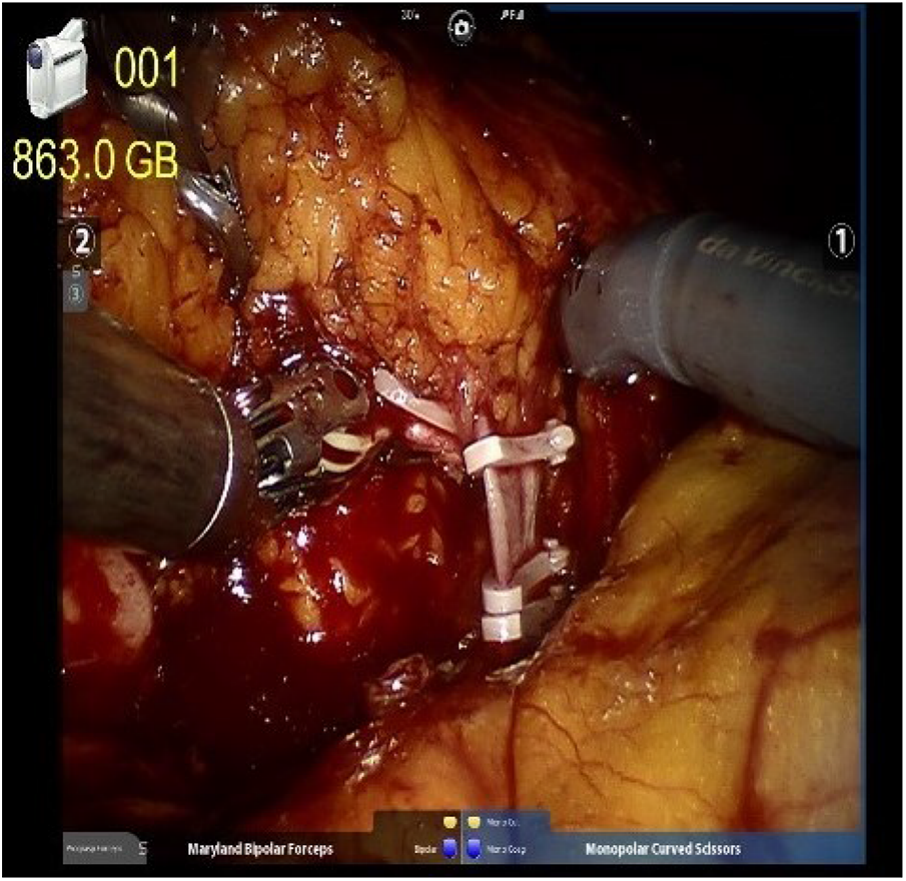
The renal artery and renal vein were double-ligated in the proximal end and single-ligated in the distal end with surgical clips.

We implemented a meticulous redocking technique for RARNU, marked by precision in the dissection across the entire ureter segment, lymph node dissection based on a defined template, precise bladder cuff excision, and a meticulous assessment of bladder closure's water-tightness. Employing the da Vinci Si robot-assisted platform by Intuitive Surgical Inc. (Sunnyvale, CA, USA), all surgical interventions were conducted under insufflation pressures ranging from 10 to 12 mmHg, utilizing a standard insufflation device. The entire procedure was executed with a minimally invasive approach involving the re-docking of the robot, a technique that preserves the range of motion for each robotic arm. A standardized step-wise transperitoneal approach is used with an extravesical excision of a bladder cuff with the ureter ligated with a Hemlock clip distal to the tumor after renal hilum controlled (Figure 1).

**Figure 2:**
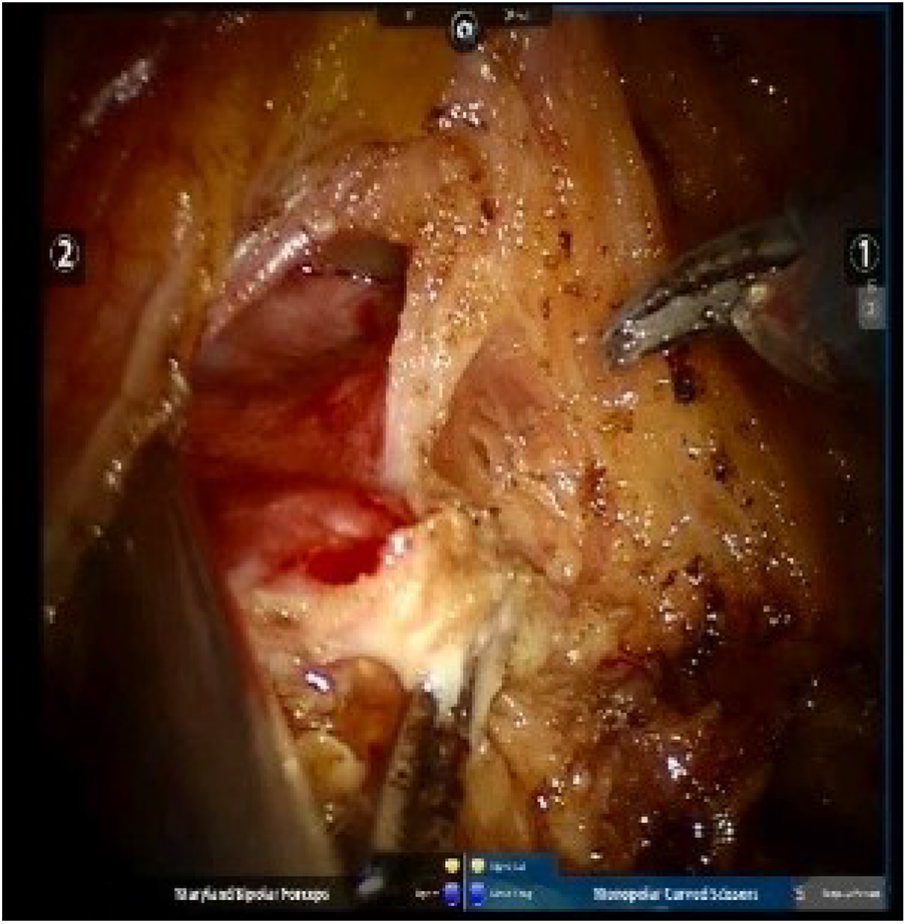
Excision of the distal ureter and bladder cuff, including the ureteric orifice, was done along the ureter.

In the intraoperative (IO) MMC group, after the ureter ligated with Hemlock clip (Teleflex Inc., Morrisville, NC, USA), intravesical mitomycin C instillation was followed as MMC 40 mg in 40 ml of sterile water was injected into the bladder through Foley catheter and then clamped. After a certain period, typically 45–60 min, the catheter is unclamped, and the chemotherapeutic agents are drained passively, followed by irrigation. Irrigation of the bladder, before excision of the distal ureter and bladder cuff, with 200–250 ml saline to avoid extravasation from the bladder with chemical toxicity to the surrounding tissue in the pelvis. During the clamping of the Foley catheter, the operation was carried on for excision of the distal ureter and bladder cuff, including the ureteric orifice (Figure 2).

**Figure 3:**
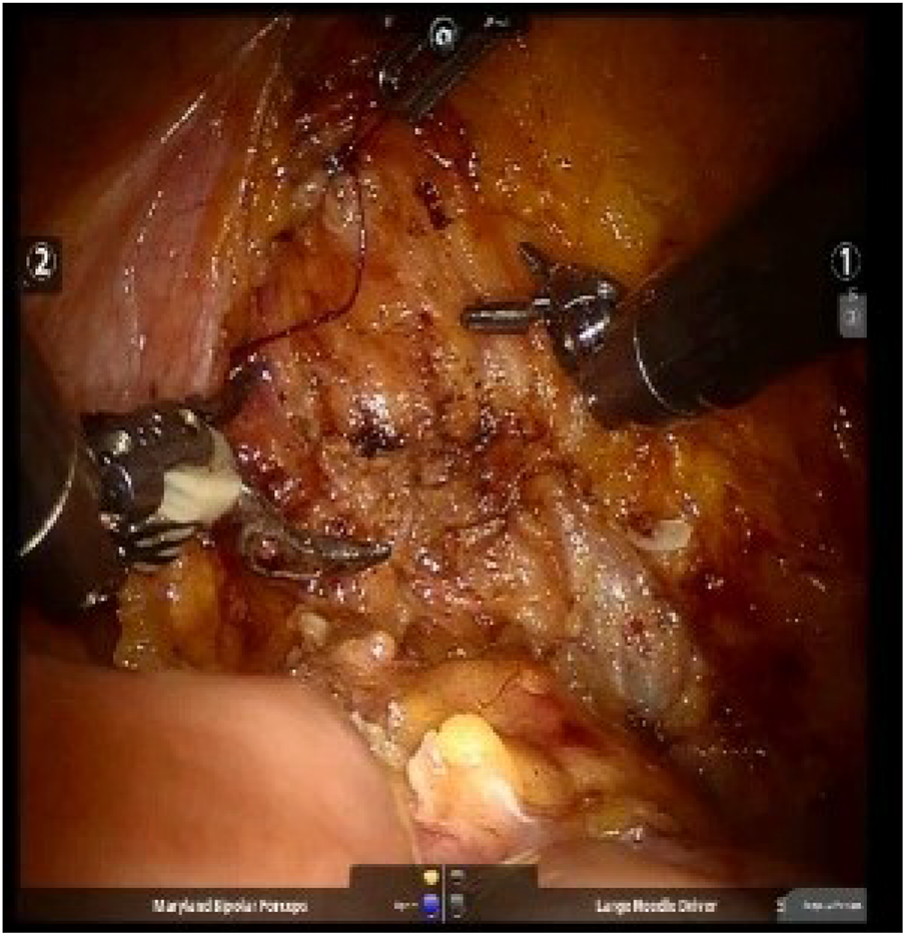
The cystotomy was closed intracorporeally with absorbable continuous sutures, followed by a bladder leak test.

The cystotomy is closed intracorporeally with an absorbable 2-0 V-Loc (Covidien, Mansfield, MA, USA) or 2-0 Quill (Westwood, MA, USA) (20 cm) continuous suture and bladder leak test is followed (Figure 3). Template-based lymph node dissection is done for every patient. The specimen is placed within an EndoCatch^TM^ bag (Covidien, Mansfield, MA, USA) and removed through an extension of the camera port. All surgeries were executed by a single surgical team under the guidance of the same surgeon.

### Outcome measures

The primary outcome was the risk of bladder recurrence and after surgery at follow-up. The secondary outcome was the occurrence of postoperative adverse events assessed by the Clavien–Dindo classification (11).

Post RARNU, surveillance for bladder recurrence encompassed cystoscopy and urine cytology conducted at three-month intervals within the initial two years, transitioning to intervals of 6–12 months thereafter. Furthermore, imaging procedures such as contrast-enhanced abdominal/pelvic CT or MRI were performed annually, alongside chest imaging (12).

### Statistical analysis

Descriptive statistics of patients' demographic and clinical characteristics are presented as number (n) and percentage (%) and performed by the chi-squared or Fisher Exact test. Continuous data with normal distribution are presented as mean ± standard deviation (SD) using Student's test; continuous data without normal distribution are presented as the median and interquartile range (IQR) and performed by Mann–Whitney *U*-test. Hazard ratios (HRs) and 95% confidence intervals (CIs) produced by Cox regression analysis were used to evaluate the associations between the study variables and the outcomes. Any variable with a *p*-value <0.2 in the univariate analysis was input in the multivariable analysis. Kaplan–Meier plot with log-rank test were depicted to demonstrate the survival outcomes between the comparison groups. The results were considered statistically significant at *p* < 0.05, and all statistical analyses were performed using the statistical package SPSS for Windows (Version 21.0, SPSS Inc., IBM Corp., Armonk, NY, USA).

## Result

### Characteristics of the study cohort

Data from 97 patients undergoing RARNU for UTUC during the study period were obtained. Of the patients, a total of 54 patients were included in the analysis after applying the inclusion and exclusion criteria. Table 1 shows the baseline demographic and clinical characteristics of 54 patients. Of whom 26 patients received MMC intraoperatively (the IO group), while 28 received deferred MMC at least 24 h postoperatively (the PO group). Patients' mean age was 75.0 ± 9.3 years, and 59.3% were females. No significant differences in presence of concomitant carcinoma-*in-situ* (CIS), surgical margin status, pathologic T (pT), pathologic grade (pGrade), multifocality, lymph vascular invasion (LVI), perineural invasion (PNI) or lymph node dissection were noted between the groups. We identified positive surgical margins as those confirmed to be margin positive in the ureters. One-year bladder recurrence rate (7.7% vs. 35.7%, *p* = 0.021) and median time to bladder recurrence (*p* = 0.034) were significantly different between the two groups.

**Table 1:** Characteristics of the study population.

	Total (*n* = 54)	IO (*n* = 26)	PO (*n* = 28)	*p*-Value
Age, years	75.0 ± 9.3	74.7 ± 9.4	75.2 ± 9.3	0.839
Sex				0.743
Male	22 (40.7)	10 (38.5)	12 (42.9)	
Female	32 (59.3)	16 (61.5)	16 (57.1)	
ASA				0.279
1	0 (0.0)	0 (0.0)	0 (0.0)	
2	14 (25.9)	5 (19.2)	9 (32.1)	
3	40 (74.1)	21 (80.8)	19 (67.9)	
4–5	0 (0.0)	0 (0.0)	0 (0.0)	
Margins				0.706^b^
Positive	8 (14.8)	3 (11.5)	5 (17.9)	
Negative	46 (85.2)	23 (88.5)	23 (82.1)	
Concomitant CIS				0.310
Present	16 (29.6)	6 (23.1)	10 (35.7)	
Absent	38 (70.4)	20 (76.9)	18 (64.3)	
Hospital stay, days	10 (9.0, 10.0)	9.5 (8.8, 10.0)	10.0 (9.0, 10.0)	0.735^a^
EBL, ml	134.3 ± 42.7	135.0 ± 43.8	133.6 ± 42.4	0.904
Clavien–Dindo grade				0.598^b^
None	46 (85.2)	24 (85.7)	22 (84.6)	
I	3 (5.6)	1 (3.6)	2 (7.7)	
II	1 (1.9)	3 (1.7)	1 (3.8)	
IIIa	0 (0.0)	0 (0.0)	0 (0.0)	
IIIb	0 (0.0)	0 (0.0)	1 (3.8)	
IVa/IVb/V	0 (0.0)	0 (0.0)	0 (0.0)	
Bladder recurrence				**0**.**021**^b^
Yes	12 (22.2)	2 (7.7)	10 (35.7)	
No	42 (77.8)	24 (92.3)	18 (64.3)	
Median time to bladder recurrence, days	365.0 (365.0, 365.0)	365.0 (365.0, 365.0)	365.0 (151.5, 365.0)	**0**.**034**^a^
LN/distant metastasis				0.353
Yes	5 (9.3)	1 (3.8)	4 (14.3)	
No	49 (90.7)	25 (96.2)	24 (85.7)	
Histology				0.868^b^
Urothelial	45 (83.3)	21 (80.8)	24 (85.7)	
Squamous	4 (7.4)	2 (7.7)	2 (7.1)	
Sarcomatoid	5 (9.3)	3 (11.5)	2 (7.1)	
pT				0.385
T0/Tis/Ta	11 (20.4)	5 (19.2)	6 (21.4)	
T1–T2	18 (33.3)	11 (42.3)	7 (25.0)	
T3–T4	25 (46.3)	10 (38.5)	15 (53.6)	
pN				**0**.**036**^b^
N0	45 (83.3)	25 (96.2)	20 (71.4)	
N1	0 (0.0)	0 (0.0)	0 (0.0)	
N2	1 (1.9)	0 (0.0)	1 (3.6)	
Nx	8 (14.8)	1 (3.8)	7 (25.0)	
pGrade				0.342^b^
High	50 (92.6)	23 (88.5)	27 (96.4)	
Low	4 (7.4)	3 (11.5)	1 (3.6)	
Multifocal				0.440
Yes	20 (37.0)	11 (42.3)	9 (32.1)	
No	34 (63.0)	15 (57.7)	19 (67.9)	
LVI				0.353^b^
Yes	5 (9.3)	1 (3.8)	4 (14.3)	
No	49 (90.7)	25 (96.2)	24 (85.7)	
PNI				0.491^b^
Yes	2 (3.7)	0 (0.0)	2 (7.1)	
No	52 (96.3)	26 (100.0)	26 (92.9)	
Lymph node dissection				1.000^b^
Yes	41 (75.9)	20 (76.9)	21 (75.0)	
No	13 (24.1)	6 (23.1)	7 (25.0)	

LN, lymph node; MMC, mitomycin-C; IO, intraoperative MMC instillation; PO, postoperative MMC instillation (at least 24 h after operation); ASA, American Society of Anesthesiologists; EBL, estimated blood loss; CIS, carcinoma *in situ*; LVI, lymph vascular invasion; PNI, perineural invasion.

*p*-value < 0.05 is shown in bold.

Continuous data with normal distribution are presented as mean ± SD; data without normal distribution are presented as median (IQR). Categorical data are presented as *n* (%).

^a^ Tested by the Mann–Whitney *U*-test.

^b^ Tested by the Fisher Exact Test.

### Risk of bladder recurrence between IO vs. PO

**Figure 4:**
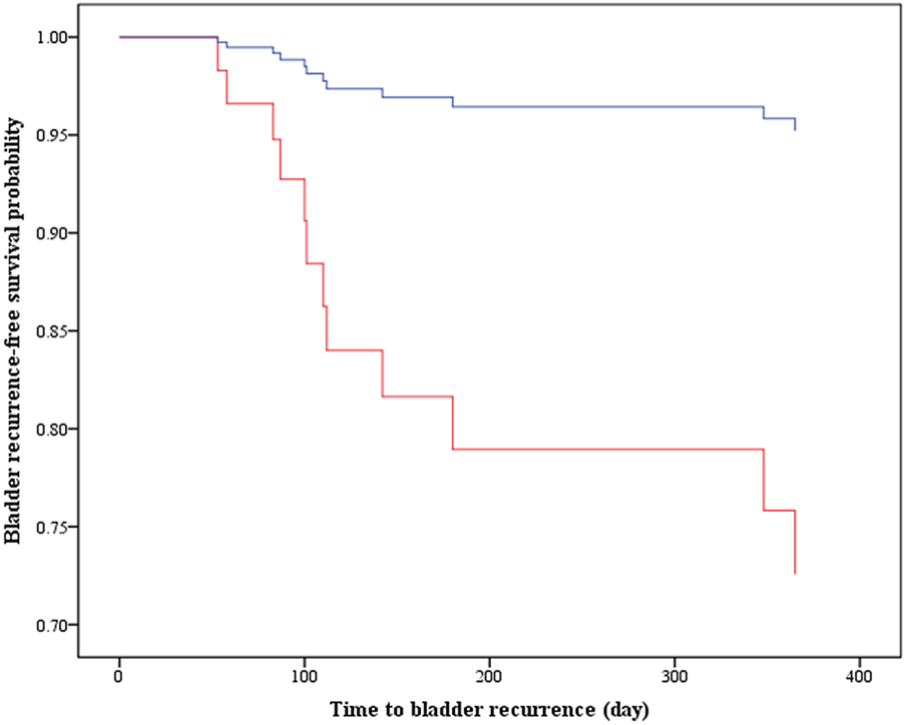
Kaplan–Meier curves display the estimated bladder recurrence-free survival. IO, intraoperative MMC instillation; PO, postoperative MMS instillation (i.e., deferred MMC at least 24 h after surgery); aHR, adjusted hazard ratio; CI, confidence interval.

Table 2 summarizes the univariate and multivariable Cox analyses on bladder recurrence. After adjusting for age, sarcomatoid, multifocal and PNI, IO remained significantly associated with a lower risk for bladder recurrence compared to PO [adjusted hazard ratio (aHR) = 0.15, 95% CI = 0.03–0.81, *p* = 0.028]. The Kaplan-Meier curves of bladder recurrence-free survival among the IO and PO groups are depicted in Figure 4.

**Table 2:** Univariate and multivariable Cox analyses for bladder recurrence.

Study variables	Univariate			Multivariable		
	HR	95% CI	*p*-Value	aHR	95% CI	*p*-Value
IO vs. PO	0.19	0.04–0.86	0.031	0.15	0.03–0.81	**0** **.** **028**
Age, years	1.06	0.99–1.15	0.117	1.05	0.95–1.15	0.352
Sex (male vs. female)	1.10	0.35–3.45	0.876			
ASA (3 vs. 2)	0.96	0.26–3.55	0.951			
Hospital stay, day	0.92	0.66–1.29	0.636			
EBL (ml)	1.00	0.99–1.01	0.999			
Margins (positive vs. negative)	2.11	0.57–7.80	0.264			
Sarcomatoid (yes vs. no)	5.08	1.36–18.93	0.016	10.25	2.14–49.19	**0** **.** **004**
Concomitant CIS (yes vs. no)	1.27	0.38–4.21	0.699			
pT (vs. T0/Tis/Ta)						
T1/T2	0.57	0.08–4.07	0.579			
T3/T4	1.93	0.41–9.10	0.405			
pGrade (high vs. low)	0.90	0.12–6.95	0.917			
Multifocal (yes vs. no)	2.61	0.83–8.24	0.101	3.90	0.91–16.77	0.067
LVI (yes vs. no)	2.28	0.50–10.43	0.287			
PNI (yes vs. no)	7.68	1.61–36.54	0.010	4.50	0.75–27.07	0.100
Lymph node dissection (yes vs. no)	0.98	0.260–3.60	0.970			

MMC, mitomycin-C; IO, intraoperative MMC instillation; PO, postoperative MMC instillation (at least 24 h after surgery); ASA, American Society of Anesthesiologists; EBL, estimated blood loss; CIS, carcinoma *in situ*; LVI, lymph vascular invasion; PNI, perineural invasion; NA, no event occurred in a classified subgroup; HR, hazard ratio; aHR, adjusted hazard ratio; CI, confidence interval.

*p*-value < 0.05 is shown in bold.

Variables with *p*-value <0.2 in the univariate analysis were adjusted for in the multivariable analysis.

Additional analysis on the factors associated with lymph node (LN)/distant metastasis showed no significant impact of MMC instillation timing (Supplementary Table S1).

### Adverse events

There were seven postoperative Clavien–Dindo Grade I or II complications, including two patients with ileus, one patient with pneumonia, two patients with sepsis, one with a wound infection and one with self-limiting delirium, all managed conservatively (Table 1). Ileus led to a prolonged stay with a median (range) of 6 (4–11) days. Only one Clavien–Dindo Grade III complication was noted as wound infection managed as debridement and delayed suture. Fortunately, there is no adverse event potentially related to MMC instillation was noted, both during the inpatient stay and at first clinic follow-up at 2–3 weeks postoperatively.

## Discussion

We retrospectively investigated the adult patients undergoing RARNU for UTUC with intraoperative MMC vs. deferred MMC instillation at our institution. The results indicate that intraoperative MMC is independently associated with a lower risk for bladder recurrence than deferred MMC administration after surgery during a 1-year follow-up. For safety issues, the intraoperative instillation group showed comparative safety profile to the deferred instillation group, with no MMC-related adverse event observed.

The occurrence of bladder recurrence after RNU is highly prevalent, thus emphasizing the necessity of routine cystoscopic surveillance, a recommendation prominently outlined in the NCCN guidelines (12, 13). The high bladder recurrence rate can be attributed to potential factors such as tumor seeding and implantation. This phenomenon involves the dissemination of malignant cells along the urinary tract lining, with these cells infiltrating downstream sites by traversing the urothelium (5, 6). Therefore, treatment strategies have focused on reducing the bladder recurrence rate after RNU, and it has been suggested that the intravesical instillation of chemotherapeutic agents significantly decreased the risk of bladder recurrence in patients with primary UTUC (5, 14–16). In theory, there is a potential for preventive action post-nephroureterectomy, as it could theoretically involve the destruction of viable seeding cells originating from the upper tract or impede the development of metachronous tumors proliferating within the bladder (5).

Adverse events after early MMC instillation still left a big concern, in which primary clinical concern was the potential extravasation of the MMC. Based on the risk of possible extravasation of MMC, the EAU guideline only suggests installing a single postoperative dose of intravesical chemotherapy, such as mitomycin C or pirarubicin, 2–10 days after surgery. The same concern is also mentioned in the ODMIT-C Trial, the first prospective and PCT trial as postoperative IVC following RNU. In that trial, the pragmatic decision was made to administer the chemotherapy when the urologist was as confident as possible that the bladder had healed and was safe to remove the Foley catheter. Thus, the timing of MMC administration showed variation between patients based on the duration of catheterization (6).

Increasing literature support that MMC instillation within the first 24 h is more effective than deferred instillation after two weeks or prolonged adjuvant MMC. This finding is consistent with the bladder tumor research, which reveals that the best time to administer the first instillation is within 24 h to prevent bladder recurrence after transurethral resection of bladder tumor (TURBT) (8). A RCT by Bosschieter J et al. found that the first instillation beginning within 24 h shows lower bladder recurrence than at the deferred group 2 weeks later and no severe adverse effects of the early instillation group (4). In general, our findings are consistent with these prior reports on the potential benefits of early MMC instillation over the deferred administration against bladder recurrence.

The prognostic significance of variant histology has been acknowledged in multiple recent studies. Upper Tract Urothelial Carcinoma (UTUC) featuring histological variants has been significantly linked to a more biologically aggressive disease, higher rates of bladder recurrence, and differing survival outcomes (17). Similarly, another study (18) provided additional evidence supporting these findings. In our current analysis, despite the small case number, we observed that sarcomatoid histology was significantly associated with increased bladder recurrence, even after adjusting for other variables. This aligns with the trends reported in previous research.

On the other hand, a previous study documented that surgical margin's location represents distinct risk factors' patterns in the setting of radical cystectomy. Concomitant CIS was associated with ureteric positive margins, while urethral and soft-tissue PSM showed worse disease-specific survival rates (19). The authors of that study suggest that clinical decision-making paradigms on adjuvant treatment and surveillance might be adapted based on positive margin and their location. In the present study, we identified positive surgical margins as those confirmed to be positive in the ureters. Nevertheless, while there appeared to be a trend, our data did not show that these positive margins were statistically significant in their association with bladder recurrence or overall survival, likely due to the limited number of patients. Further research involving a larger cohort is necessary to validate our conclusions.

### Limitation

Several limitations of this study should be noted. Firstly, our study did not employ random allocation; instead, the study's treatment allocation was time-based, as documented previously. This approach could introduce selection bias. Secondly, the study does not take into account financial considerations, and the substantial expense associated with robotic surgery could render it inaccessible to many patients. This could also potentially introduce selection bias into the analytic results. Thirdly, it is understood that IO might increase the length of surgical procedures; however, due to the lack of data collection on operative times, an analysis could not be conducted.

## Conclusion

Intraoperative intravesical MMC instillation during RNU is safe and associated with lower bladder recurrence risk compared to deferred instillation postoperatively. Future large, prospective studies are still needed to confirm the current findings.

## Data Availability

The original contributions presented in the study are included in the article/**Supplementary Material**, further inquiries can be directed to the corresponding author.
