# Supplementary material for: Safety and oncological outcome of early intraoperative intravesicle mitomycin C vs. deferred instillation in patients receiving robot-assisted radical nephroureterectomy
Source: Front Surg. 2024 Apr 25;11:1366982. doi: 10.3389/fsurg.2024.1366982 (PMC11079113; doi:10.3389/fsurg.2024.1366982)
Supplement: Supplementary file 1 [file Table1.docx]

| **Supplementary Table 1. Univariate and** **multivariable Cox analyses for LN or distant metastasis.** | | | | | | |
| --- | --- | --- | --- | --- | --- | --- |
| **Variable** | **Univariate** | | | **Multivariable** | | |
|  | **HR** | **95% CI** | **p value** | **aHR** | **95% CI** | **p value** |
| **IO vs. PO** | 0.27 | 0.03 - 2.38 | 0.236 | 0.40 | 0.03 - 4.66 | 0.462 |
| **Age** | 1.04 | 0.93 - 1.16 | 0.504 |  |  |  |
| **Sex (male vs. female)** | 0.35 | 0.04 - 3.15 | 0.350 |  |  |  |
| **ASA (3 vs. 2)** | 0.50 | 0.08 - 2.99 | 0.447 |  |  |  |
| **Hospital stay, day** | 0.82 | 0.44 - 1.52 | 0.533 |  |  |  |
| **EBL (ml)** | 1.00 | 0.99 - 1.01 | 0.954 |  |  |  |
| **Margins (positive vs. negative)** | 4.02 | 0.67 - 24.10 | 0.127 | 1.62 | 0.2 - 13.44 | 0.655 |
| **Sarcomatoid (yes vs. no)** | 9.13 | 1.51 - 55.14 | **0.016** | 29.73 | 2.62 - 337.47 | **0.006** |
| **Concomitant CIS (yes vs. no)** | 0.58 | 0.07 - 5.22 | 0.630 |  |  |  |
| **pT (vs. T0/Tis/Ta)** |  |  |  |  |  |  |
| T1/T2 | NA | NA | NA |  |  |  |
| T3/T4 | 3.70 | 0.62 - 22.12 | 0.152 |  |  |  |
| **pGrade (high vs. low)** | 22.69 | 0 - 45416744.29 | 0.673 |  |  |  |
| **Multifocal (yes vs. no)** | 1.10 | 0.18 - 6.59 | 0.915 |  |  |  |
| **LVI (yes vs. no)** | 2.35 | 0.26 - 21.01 | 0.445 |  |  |  |
| **PNI (yes vs. no)** | 19.05 | 3.14 - 115.55 | **0.001** | 29.85 | 2.08 - 428.29 | **0.012** |
| **Lymph node dissection (yes vs. no)** | 0.49 | 0.08 - 2.93 | 0.434 |  |  |  |
| Abbreviation: MMC, mitomycin-C; IO, intraoperative MMC; PO, postoperative MMC instillation (at least 24 hours after surgery); ASA, American Society of Anesthesiologists; EBL, estimated blood loss; CIS, carcinoma in situ; NA, no event occurred in a classified subgroup; HR, hazard ratio; aHR, adjusted hazard ratio; CI, confidence interval.  P-value < 0.05 is shown in bold.  Variables with p-value < 0.2 in the univariate analysis were adjusted for in the multivariable analysis. | | | | | | |
